# Supplementary material for: Changes in Adenosine Deaminase Activity and Endothelial Dysfunction after Mild Coronavirus Disease-2019
Source: Int J Mol Sci. 2023 Aug 24;24(17):13140. doi: 10.3390/ijms241713140 (PMC10487738; doi:10.3390/ijms241713140)
Supplement: Supplementary file 1 [file ijms-24-13140-s001.zip › ijms-2554981-supplementary.pdf]

Circulating and endothelial adenosine deaminase after mild COVID-19—A possible role in microvascular complications

Supplementary Material Online

Parameter	Controls (n=5)
Age (years)	35.6 ± 1.53
Female	n=3 (60%)
BMI (kg/m2)	23.2 ± 1.39

Table S1. Characteristics of healthy controls without prior COVID-19 diagnosis included in microcirculation function study using FMSF technique.

Parameter	Control (n=25)	postCOVID (n=40)
Alanine	269 ± 14	267 ± 10
Asparagine	41.7 ± 1.9	39.8 ± 1.4
Aspartate	11.8 ± 0.9	11.1 ± 0.8
Cystine	19.5 ± 2.4	11.7 ± 1.0**
Glutamate	166 ± 10	246 ± 9.3****
Glutamine	719 ± 24	891 ± 24****
Glycine	276 ± 19	214 ± 9.3**
Histidine	78.8 ± 2.5	78.2 ± 1.6
Isoleucine	119 ± 6.2	137 ± 7.4
Leucine	115 ± 4.8	120 ± 5.2
Lysine	170 ± 5.2	190 ± 3.7**
Methionine	24.2 ± 1.3	29.2 ± 1.7*
Phenylalanine	114 ± 3.7	112 ± 2.6
Proline	157 ± 12	164 ± 12
Serine	151 ± 9.4	154 ± 11
Threonine	144 ± 7.9	157 ± 8.4
Tryptophan	51.2 ± 3.6	52.3 ± 2.1
Tyrosine	99.2 ± 6.7	109 ± 7.5
Valine	370 ± 16	462 ± 14****
1-methylhistidine	3.82 ± 0.2	4.00 ± 0.2
3-methylhistidine	2.43 ± 0.4	3.13 ± 0.3
Betaine	36.1 ± 1.6	39.5 ± 1.7
Sarcosine	8.42 ± 0.4	8.83 ± 0.4
Taurine	78.3 ± 6.5	87.5 ± 4.1

Table S2. Serum amino acids concentration [μmol/L] in postCOVID patients (n=40) and controls (n=25). Results are shown as mean ± SEM.*p<0.05, **p<0.01, ****p<0.0001 by Student t-test. Serum amino acids concentration [μmol/L] in postCOVID patients (n=40) and controls (n=25). Results are shown as mean ± SEM.*p<0.05, **p<0.01, ****p<0.0001 by Student t-test.
